# Environmental perceptions as mediators of the relationship between the objective built environment and walking among socio-economically disadvantaged women

**DOI:** 10.1186/1479-5868-10-108

**Published:** 2013-09-19

**Authors:** Delfien Van Dyck, Jenny Veitch, Ilse De Bourdeaudhuij, Lukar Thornton, Kylie Ball

**Affiliations:** 1 Research Foundation Flanders, Egmontstraat 5, 1000 Brussels, Belgium; 2 Department of Movement and Sports Sciences, Ghent University, Watersportlaan 2, 90000 Ghent, Belgium; 3 Centre for Physical Activity and Nutrition Research, School of Exercise and Nutrition Sciences, Deakin University, 221 Burwood Highway, Burwood, Victoria 3125, Australia

**Keywords:** Physical activity, Adults, GIS, Physical environment

## Abstract

**Background:**

Women living in socio-economically disadvantaged neighbourhoods are at increased risk for physical inactivity and associated health outcomes and are difficult to reach through personally tailored interventions. Targeting the built environment may be an effective strategy in this population subgroup. The aim of this study was to examine the mediating role of environmental perceptions in the relationship between the objective environment and walking for transportation/recreation among women from socio-economically disadvantaged neighbourhoods.

**Methods:**

Baseline data of the Resilience for Eating and Activity Despite Inequality (READI) study were used. In total, 4139 women (18–46 years) completed a postal survey assessing physical environmental perceptions (aesthetics, neighbourhood physical activity environment, personal safety, neighbourhood social cohesion), physical activity, and socio-demographics. Objectively-assessed data on street connectivity and density of destinations were collected using a Geographic Information System database and based on the objective z-scores, an objective destinations/connectivity score was calculated. This index was positively scored, with higher scores representing a more favourable environment. Two-level mixed models regression analyses were conducted and the MacKinnon product-of-coefficients test was used to examine the mediating effects.

**Results:**

The destinations/connectivity score was positively associated with transport-related walking. The perceived physical activity environment mediated 6.1% of this positive association. The destinations/connectivity score was negatively associated with leisure-time walking. Negative perceptions of aesthetics, personal safety and social cohesion of the neighbourhood jointly mediated 24.1% of this negative association.

**Conclusion:**

For women living in socio-economically disadvantaged neighbourhoods, environmental perceptions were important mediators of the relationship between the objective built environment and walking. To increase both transport-related and leisure-time walking, it is necessary to improve both objective walkability-related characteristics (street connectivity and proximity of destinations), and perceptions of personal safety, favourable aesthetics and neighbourhood social cohesion.

## Background

Despite the well-known benefits of engaging in sufficient physical activity (i.e. at least 150 minutes of moderate-to-vigorous physical activity per week) [1], large proportions of adults living in developed countries are insufficiently active. Ecological models of physical activity behaviour [2,3] emphasize the importance of studying correlates on multiple levels – individual, social, physical environmental and policy. Furthermore, ecological models posit that correlates are behaviour-specific, so research should focus on specific physical activity behaviours. Determining the most pertinent correlates of both walking for leisure and walking for transport is important to inform effective interventions aimed at increasing these behaviours. Environmental correlates may be particularly relevant as their implementation can have immediate, population-wide, benefits.

Physical or built environmental attributes related to walkability (i.e. high street connectivity, mixed land use, high residential density) have been consistently associated with active transportation in adults [4,5]. Mixed evidence has been found on the role of these traditional walkability characteristics in explaining leisure-time physical activity [6-8]. Whilst it is likely that the environment is still a relevant factor in promoting these behaviours, other physical environmental attributes like access (i.e. availability and proximity) to recreation facilities or neighbourhood aesthetics (e.g. presence of trees, absence of litter, attractive scenery) may be more essential in influencing leisure-time walking and moderate-to-vigorous physical activity [9].

It is particularly important to examine the contribution of the physical environment to explain physical activity in individuals with low educational levels or those living in socio-economically disadvantaged neighbourhoods and in women of childbearing age (18–46 years). These population subgroups are at increased risk for physical inactivity and associated outcomes including overweight or obesity [10,11]. Furthermore, it is known that individuals living in low-income neighbourhoods are difficult to reach through personally tailored interventions [12] and that women with children possibly have strong barriers towards physical activity (e.g. lack of time, lack of energy, childcare issues) [13]. Thus, targeting the physical environment might be a more effective strategy for achieving behaviour change in these target groups.

To assess environmental characteristics related to neighbourhood walkability, both objective and self-report measurement methods can be used. Reports of the level of concordance between the objective and perceived environment have been mixed [14-16], so it remains unclear how strong the agreement between both types of measures is. Furthermore, it is possible that objectively measured high-walkable environments, characterized by high residential density, mixed land use and high street connectivity and typically situated in city centres, induce some negative environmental attributes, like crime- and traffic-related safety problems, poor-maintained buildings and litter. A previous Belgian study confirmed this hypothesis, showing that adults living in objectively-assessed high walkable neighbourhoods perceived more aesthetic-related problems, pollution, crime and less overall safety than those living in low-walkable neighbourhoods [17].

It remains unknown how a mix of positive and negative environmental characteristics may affect physical activity and whether these associations work directly or indirectly (mediations). Several theoretical frameworks, like the ecological models [2,3] and the ENrG framework [18] emphasize the importance of examining cognitive factors (e.g. intrapersonal factors, environmental perceptions) as potential mediators of the relationship between the objective physical environment and physical activity. However, until now these potential mediators have rarely been included in public health research. One previous Canadian study found evidence of clustering of environmental characteristics: adults living in a high-walkable neighbourhood with few green spaces did more active transportation than those living in a low-walkable neighbourhood with many green spaces [19]. This suggests that walkability characteristics are of overriding importance to explain transport-related walking. To our knowledge, no other studies have examined these clustered associations, nor the mediating effects of environmental perceptions in explaining the relationship between the objective environment and physical activity. Examining these mediating effects can help to better understand the relationship between the objective/perceived environment and physical activity and can help to identify which factors to focus on when developing programs to optimize the built environment.

Based on the shortcomings in the current literature, the aim of the present study was to examine the mediating effects of specific environmental perceptions in the relationship between objective environmental characteristics and walking for transportation/recreation in women from socio-economically disadvantaged neighbourhoods. The current study focused on walking, because this is the most common and accessible type of physical activity in adults having documented health benefits [20] and because the impact of the neighbourhood environment is supposed to be strongest for walking [4].

## Methods

For this study, baseline data of the Resilience for Eating and Activity Despite Inequality (READI) study were used. Details on the study protocol of this study have been published elsewhere [21]. Briefly, the READI study is a longitudinal cohort study examining resilience to obesity among women (18–46 years) and their children (5–12 years) living in socio-economically disadvantaged neighbourhoods. Baseline data collection took place between August 2007 and January 2008. The study protocol was approved by the Deakin University Human Research Ethics Committee, the Victorian Department of Education and the Catholic Education Office.

### Procedure and participants

Participants were randomly selected using the electoral roll (registration is compulsory for Australian citizens) from 40 rural and 40 urban socially and economically disadvantaged suburbs in Victoria, Australia. To select the 80 READI suburbs, all suburbs in Victoria were categorized into low-, middle- or high-disadvantage tertiles, using the Australian Bureau of Statistics’ Socio-economic Index for Areas (SEIFA). The SEIFA is an area-based measure of socio-economic disadvantage, constructed from the population census [22]. Suburbs in the lowest SEIFA tertile that had populations of ≥1200 people and were located within 200 km of Melbourne, formed the READI suburb selection pool. In total, 50 women aged 18–45 years from each suburb were selected (or, where there were fewer than 150 women in this age range living in the suburb, all women within the age range were selected).

In total, 11940 women living in these suburbs were posted a survey, assessing a broad range of factors that might influence women’s lifestyle choices associated with obesity risk. Two reminder letters (10 days and 20 days after initially mailing the survey) were sent to those who did not return their survey [23]. Of the surveys distributed, 861 were returned as undeliverable and 17 were returned from individuals who were not eligible (e.g. men). In total, 4934 completed surveys were returned (45% response rate). Of these 4934 women, 571 were excluded because they no longer lived in a READI suburb, nine were excluded because they were not within the desired age range (18–46 years), three were excluded because the survey was not completed by the person it was addressed to, and two subsequently requested to be withdrawn from the study. Because physical activity was examined as an outcome measure in the present study, only women who were not pregnant at the time of the survey were included in the analyses. Applying this exclusion criterion led to a final sample of 4139 women. The mean number of participants per suburb was 52 (SD: 9), ranging from 29 to 72 across suburbs.

### Measures

#### Objectively-assessed physical environment

Data on the spatial location of amenities within a 2 km pedestrian road network buffer [24] around each participant’s residence were collected using multiple secondary sources including online directories, commercially available datasets, and state and local government spatial datasets. These data were mapped using a Geographic Information System (GIS) (ArcGIS 9.3). Characteristics of density of physical activity/food related destinations and street connectivity were assessed, because these walkability-related characteristics have previously been shown to be associated with walking among adults [7]. To assess the density of physical activity/food related destinations, the number of fast food restaurants (e.g. Pizza Hut, McDonalds, Nandos, Subway), supermarkets (major chain, other supermarkets and green grocers), sports facilities (gym/leisure centres and public swimming pools) and playgrounds within the 2 km buffer were counted. Z-scores were calculated for each separate destination and all z-scores were summed to obtain an overall ‘destinations’ score. Street connectivity was defined as the number of three- or more-way intersections within the 2 km buffer and a standardized z-score was calculated [25]. Finally, the objective ‘destinations/connectivity’ z-score was calculated by summing z-scores for density of physical activity/food related destinations and street connectivity.

#### Perceived environment

Existing measures were used to assess perceptions of aesthetics (mean of five items, Cronbach’s alpha = 0.74), the neighbourhood physical activity environment (mean of seven items, Cronbach’s alpha = 0.80), personal safety (mean of three items, Cronbach’s alpha = 0.85) [26] and social cohesion of the neighbourhood (mean of five items, Cronbach’s alpha = 0.83) [27]. The different scales, items and response categories are presented in Table 1.

**Table 1:** Construction of the items and scales to assess the perceived physical environment

	**Content of the items**	**Response category**
Aesthetics (five items)	1. There is a lot of rubbish on the streets in my neighbourhood	Five point scale, from strongly disagree to strongly agree
	2. There is a lot of noise in my neighbourhood	
	3. In my neighbourhood the buildings and homes are well-maintained	
	4. The buildings and homes in my neighbourhood are interesting	
	5. My neighbourhood is attractive	
Neighbourhood PA environment (seven items)	1. My neighbourhood offers many opportunities to be physically active	Five point scale, from strongly disagree to strongly agree
	2. Local sports clubs and other facilities in my neighbourhood offer many opportunities to get exercise	
	3. It is pleasant to walk in my neighbourhood	
	4. The trees in my neighbourhood provide enough shade	
	5. In my neighbourhood it is easy to walk places	
	6. I often see other people walking in my neighbourhood	
	7. I often see other people exercising in my neighbourhood	
Personal safety (three items)	1. I feel safe walking in my neighbourhood, day or night	Five point scale, from strongly disagree to strongly agree
	2. Violence is not a problem in my neighbourhood	
	3. My neighbourhood is safe from crime	
Neighbourhood social cohesion (five items)	1. People in my neighbourhood can be trusted	Five point scale, from strongly disagree to strongly agree
	2. This is a close-knit neighbourhood	
	3. People around here are willing to help their neighbours	
	4. People in this neighbourhood generally do not get along with each other (reverse scored)	
	5. People in this neighbourhood do not share the same values (reverse scored)	

*PA* physical activity.

#### Self-reported physical activity

Self-reported physical activity was collected using the long version of the International Physical Activity Questionnaire (IPAQ-L). The IPAQ is suitable for use in adults aged 15–69 years, has excellent test-retest reliability properties (pooled r = 0.81) and acceptable validity (mean ρ = 0.30) when compared with accelerometer-based physical activity [28]. Frequency (number of days in the last seven days) and duration (hours and minutes per day) of physical activity in different domains (work, transportation, recreation and household) were assessed. Minutes/week of different types of physical activity were calculated by multiplying frequency with duration. Only min/week of walking for transportation and min/week of leisure-time walking were used in the present analyses.

#### Socio-demographic covariates

Possible covariates were selected based on evidence in previous literature examining associations of socio-demographic characteristics with transport-related and leisure-time walking [29]. Self-reported information on participants’ age, educational level (low: <Year 12; medium: Year 12/trade/certificate; High: university/postgraduate), employment status (employed: including full-time work and part-time work; unemployed: including those unemployed/laid off, keeping house/raising children, full time study and retired), smoking status (yes; no), marital status (married/in a relationship; alone, including separated, divorced, widowed; and never married), number of children (none; one or more) residential location (urban; rural) and BMI were collected. Only those variables that correlated significantly with transport-related and/or leisure-time walking were included as covariates in the statistical analyses. Consequently, age, educational level, employment status, smoking status and marital status were included as covariates in all analyses.

### Statistical analyses

Analyses were conducted using SPSS 19.0. The walking variables were positively skewed, therefore, the square root of the original variables was used in the analyses to improve normality. Raw data were used to calculate the descriptive statistics, shown in Table 2.

**Table 2:** Socio-demographic sample characteristics, walking behaviour and objective/perceived physical environmental characteristics

**Variable**	**Total sample**
**Socio-demographic characteristics**	
Age (mean [SD]; *n = 4083*)	34.6 (8.2)
Educational level (%; *n = 4073*)	
Low	22.6
Medium	51.5
High	25.9
Employment status (%; *n = 4129*)	
Employed	68.1
Unemployed	31.9
Smoking status (%; *n = 4136*)	
Smoker	25.7
Non-smoker	74.3
Marital status (%; *n = 4112*)	
Married/in a relationship	64.3
Alone	35.7
Number of children up to 18 years in household (%; *n = 4056*)	
None	39.4
One	17.6
Two	26.1
Three or more	17.0
Residential location (%; *n = 4139*)	
Urban	46.6
Rural	53.4
Body Mass Index in kg/m^2^ (mean [SD]; *n = 3868*)	25.9 (5.9)
**Walking behaviour (mean [SD])**	
Walking for transportation (min/week; *n = 4036*)	176.6 (277.4)
Leisure-time walking (min/week; *n = 4070*)	114.8 (173.2)
**Objectively-assessed physical environment (2 km network buffer; mean [SD]; *n = 4125*)**	
Number of fast food restaurants	1.8 (3.1)
Number of supermarkets/grocery stores	4.8 (10.8)
Number of sports facilities	1.0 (1.3)
Number of playgrounds	7.5 (5.9)
Number of 3+ leg intersections	217.6 (165.5)
Standardized ‘destinations/connectivity’ z-score^b^	0.01 (4.03)
**Perceived environment (mean [SD])**^**a**^	
Aesthetics, mean of 5 items (*n = 4085*)	3.7 (0.6)
Neighbourhood physical activity environment, mean of 7 items (*n = 4069*)	3.7 (0.6)
Personal safety, mean of 3 items (*n = 4099*)	3.2 (1.0)
Social cohesion of the neighbourhood, mean of 5 items (*n = 4122*)	3.3 (0.6)

*SD* standard deviation.

^a^positively scored on a 5-point Likert scale (1–5).

^b^destinations/connectivity z-score = z-fast food restaurants + z-supermarkets + z-sports facilities + z-playgrounds + z-intersections.

In all analyses, clustering of participants at suburb level was taken into account by conducting multilevel analyses (mixed models analyses in SPSS taking into account two levels: level 1 = individual; level 2 = suburb). In the first stage of the analyses, the main associations between the objective physical environment (destinations/connectivity z-score) and the outcome measures (walking for transportation and leisure-time walking) (τ), were tested. Then, in a second stage, the mediating role of different environmental perceptions on these associations was tested using the product-of-coefficients test of MacKinnon and colleagues [30]. This specific test was used as it provides greater statistical power than other commonly used mediating methods [31]. The test includes (1) estimating the effects of the destinations/connectivity z-score on potential mediators (perceived aesthetics, physical activity environment, personal safety, and social cohesion of the neighbourhood) by regressing the potential mediators onto the z-score (α-coefficients); (2) estimating the independent effect of the potential mediators on the physical activity outcome (transport-related walking or leisure-time walking) by regressing the outcome onto the destinations/connectivity z-score and the potential mediators (β-coefficients); (3) computation of the product of the two coefficients (αβ), representing the mediated effect. The statistical significance of the mediated effect was estimated by dividing the product-of-coefficient by its standard error. Moreover, the proportion mediated was calculated by dividing the product-of-coefficient (αβ) by the total main effect of the destinations/connectivity z-score on the respective outcome measure (τ of first stage of analyses). For all analyses, 95% confidence intervals (CI) were reported.

## Results

Socio-demographic characteristics, descriptive statistics of the physical activity behaviour of the sample, as well as objective and perceived physical environmental characteristics are presented in Table 2. Participants walked on averaged 176.6 (SD: 277.4) min/week for transportation, and 114.8 (SD: 173.2) min/week for recreation. Transport-related walking ranged from 118.9 (SD: 207.4) min/week to 283.3 (SD: 275.6) min/week across suburbs, while leisure-time walking ranged from 54.1 (SD: 68.2) to 187.9 (SD: 233.9) min/week.

### Main effects of the destinations/connectivity z-score on the outcome measures (τ)

In the total study sample, the destinations/connectivity z-score was positively associated with walking for transportation (β [SE] = 0.16 [0.04]; CI = 0.09, 0.23) and negatively associated with leisure-time walking (β [SE] = −0.09 [0.03]; CI = −0.16, -0.02).

### Associations between the destinations/connectivity z-score and potential mediators (α-coefficients)

The α-coefficients are presented in Tables 3 and 4. The objective destinations/connectivity z-score was negatively associated with perceived aesthetics (CI = −0.03, -0.01), personal safety (CI = −0.05, -0.03) and social cohesion of the neighbourhood (CI = −0.03, -0.01), while it was positively related to the perceived physical activity environment (CI = 0.001, 0.02). So, for all potential mediators, β-coefficients were calculated.

**Table 3:** Regression analyses for possible mediators of the associations between the destinations/connectivity score and walking for transportation

**Possible mediators**	**α (SE)**	**95% CI for α**	**β (SE)**	**95% CI for β**	**αβ (SE)**	**95% CI for αβ**	**Proportion mediated (%)**
Aesthetics	**−0.02 (0.005)**	−0.03, -0.01	0.34 (0.23)	−0.12, 0.80			
Physical activity environment	**0.01 (0.004)**	0.001, 0.02	**0.97 (0.23)**	0.52, 1.42	**0.01 (0.005)**	0.001, 0.02	**6.1**
Personal safety	**−0.04 (0.007)**	−0.05, -0.03	**0.38 (0.16)**	0.08, 0.69	**−0.02 (0.007)**	−0.03, -0.002	S
Neighbourhood social cohesion	**−0.02 (0.004)**	−0.03, -0.01	0.26 (0.24)	−0.22, 0.73			

*SE* standard error, *CI* confidence interval, *S* suppression effect.

All significant associations are presented in bold font.

*Notes:* α–coefficients were estimated by regressing the potential mediators onto the destinations/connectivity z-score; β-coefficients were estimated by regressing walking for transportation onto the destinations/connectivity z-score and potential mediators; αβ-coefficients represent the mediated effect; all possible mediators were positively scored (higher score = better perceptions); all analyses were controlled for individual-level age, smoking status, marital status, employment status and educational level.

**Table 4:** Regression analyses for possible mediators of the associations between the destinations/connectivity score and leisure-time walking

**Possible mediators**	**α (SE)**	**95% CI for α**	**β (SE)**	**95% CI for β**	**αβ (SE)**	**95% CI for αβ**	**Proportion mediated (%)**
Aesthetics	**−0.02 (0.005)**	−0.03, -0.01	**0.54 (0.20)**	0.15, 0.94	**−0.01 (0.005)**	−0.02, -0.001	**12.0**
Physical activity environment	**0.01 (0.004)**	0.001, 0.02	**1.44 (0.20)**	1.05, 1.82	**0.01 (0.006)**	0.002, 0.03	S
Personal safety	**−0.04 (0.007)**	−0.05, -0.03	**0.45 (0.13)**	0.19, 0.71	**−0.02 (0.006)**	−0.03, -0.006	**20.0**
Neighbourhood social cohesion	**−0.02 (0.004)**	−0.03, -0.01	**0.60 (0.21)**	0.19, 1.00	**−0.01 (0.005)**	−0.02, -0.003	**13.3**
**Total multiple model**					**−0.02 (0.01)**	−0.04, -0.001	**24.4**

*SE* standard error, *CI* confidence interval, *S* suppression effect.

All significant associations are presented in bold font.

*Notes:* α–coefficients were estimated by regressing the potential mediators onto the destinations/connectivity z-score; β-coefficients were estimated by regressing leisure-time walking onto the destinations/connectivity z-score and potential mediators; αβ-coefficients represent the mediated effect; all possible mediators were positively scored (higher score = better perceptions); all analyses were controlled for individual-level age, smoking status, marital status, employment status and educational level.

### Associations between potential mediators and the outcome measures (β-coefficients)

The results for transport-related walking are shown in Table 3. After taking into account the destinations/connectivity z-score, the perceived physical activity environment (CI = 0.52, 1.42) and personal safety (CI = 0.08, 0.69) were positively associated with walking for transportation. Aesthetics (CI = −0.12, 0.80) and social cohesion of the neighbourhood (CI = −0.22, 0.73) were not related to the outcome measure.

In relation to leisure-time walking (Table 4), perceived aesthetics (CI = 0.15, 0.94), the physical activity environment (CI = 1.05, 1.82), personal safety (CI = 0.19, 0.71) and social cohesion of the neighbourhood (CI = 0.19, 1.00) showed a positive association after taking into account the objective destinations/connectivity z-score.

### Mediated effects of environmental perceptions on the associations between the destinations/connectivity z-score and the outcome measures (αβ-coefficients)

The perceived physical activity environment (6.1%; CI = 0.001, 0.02) mediated the association between the objective destinations/connectivity z-score and walking for transportation (Table 3). A suppression effect for personal safety was found; which indicates that including this mediator strengthened the association between the destinations/connectivity z-score and transport-related walking.

Perceived aesthetics (12.0%; CI = −0.02, -0.001), personal safety (20.0%; CI = −0.03, -0.006) and social cohesion of the neighbourhood (13.3%; CI = −0.02, -0.003) mediated the association between the destinations/connectivity z-score and leisure-time walking (Table 4). For the perceived physical activity environment, a suppression effect was found. The multiple mediation model showed that the joint mediating effect of all environmental perceptions was significant (CI = −0.04, -0.001) and that 24.4% of the association between the destinations/connectivity z-score and leisure-time walking was mediated by all mediators together. This proportion is lower than the sum of the proportions mediated by separate mediators because of the significant correlations between different mediators (correlation coefficients ranging from r = 0.38 to r = 0.55, all p < 0.001).

## Discussion

The present study revealed several direct and mediating associations between the objective physical environment and different types of walking among Australian women living in socio-economically disadvantaged neighbourhoods. After controlling for socio-demographic covariates, the objective score including measures of density of physical activity/food related destinations and street connectivity, was positively associated with walking for transport. Previous research consistently found a positive relationship between walkability-related environmental characteristics and active transportation in adults [4,5]. The objective destinations/connectivity score was negatively associated with leisure-time walking. We are unaware of previous studies reporting negative associations between walkability-related features and leisure-time walking in adults. Hitherto, previous findings have been inconsistent with results ranging from positive associations to no significant associations [4,7,32,33]. However, no prior studies have examined these relationships among women living in socio-economically disadvantaged neighbourhoods, so the present findings could be specific for this population subgroup.

The mediating analyses presented in this paper helped to explain the negative association between the objective destinations/connectivity score and leisure-time walking. Living in an environment with high street connectivity and many destinations nearby was related to having positive perceptions of the physical activity environment, but also with perceiving poor aesthetics, low personal safety and low social cohesion of the neighbourhood. In total, 24.4% of the negative association between the objective destinations/connectivity score and leisure-time walking was explained by these negative perceptions. The present findings confirm the hypothesis that high (objective) walkability is not necessarily accompanied by positive perceptions of related environmental attributes [17]. Furthermore, it seems that these negative perceptions might outweigh the importance of walkability-related characteristics to explain specific behaviours like leisure-time walking. Living in a poor-connected neighbourhood with few destinations nearby, but perceiving high safety and favourable aesthetics may be more beneficial for leisure-time walking than living in an objectively well-connected environment with many destinations.

To explain transport-related walking, favourable objective street connectivity and density of destinations, in combination with positive perceptions of the physical activity environment seem to be of overriding importance, even when poor aesthetics and low personal safety were perceived. For transport-related walking, the present findings are in line with the results of McCormack and colleagues [19], who suggested that walkability characteristics are more important to explain active transportation than other environmental features, like the availability of green spaces.

When considering the mediating effects in more detail, it seems that objective walkability-related characteristics (i.e. density of destinations and connectivity) are directly associated with transport-related walking, while the relationship with leisure-time walking is more complex. A possible explanation for this finding could be that leisure-time walking is a type of physical activity that is consciously practiced, and governed by choice rather than necessity [3,34,35]. Probably adults, and more specifically women living in socio-economically disadvantaged neighbourhoods, do not want to walk for recreation in an environment that is aesthetically unappealing, unsafe and has a low level of social cohesion. Because transport-related walking is rather governed by necessity, it is plausible that negative perceptions of neighbourhood social cohesion, personal safety and aesthetics will not prevent women living in socio-economically disadvantaged neighbourhoods from walking for transportation.

Perceived personal safety and aesthetics have been previously related to leisure-time walking, while the perceived physical activity environment has been associated with active transportation [4,5,36]. Consequently, although the direction of some of the mediating effects was surprising, the mediating effects of these attributes per se were not unexpected. However, an important novelty of this study is the mediating effect of neighbourhood social cohesion. To our knowledge, the role of neighbourhood social cohesion to explain the relationship between the physical environment and physical activity has not been studied previously. The mediating effect identified here is a key finding for future research because social cohesion has been identified as an important factor positively related to neighbourhood satisfaction and overall physical and mental health [37-39]. Therefore, from a broader health perspective, it is necessary to further clarify these associations and to find out how feelings of social cohesion among women and other residents living in high-walkable environments can be improved.

Acknowledging the cross-sectional study design and the need for confirmation of causal relationships, it seems that to increase both transport-related and leisure-time walking, it is necessary to find an optimal balance between several environmental attributes. Walkability-related characteristics, perceptions of personal safety, favourable aesthetics, high neighbourhood social cohesion and possibly other features that were not included in this study should be optimally balanced in order to support walking. Other physical and social environmental perceptions (e.g. social support, perceived accessibility of facilities) should be included as possible mediators in future studies. This may help to fully understand the indirect nature of the relationship between objective physical environmental characteristics and walking for different purposes in socio-economically disadvantaged women.

A strength of the present study was the large study sample of women living in socio-economically disadvantaged neighbourhoods, a population subgroup being at high risk for overweight and other health problems and difficult to reach in research studies and interventions. Second, women living in both urban and rural regions were included in the study. Third, powerful mediation techniques were used in the analyses, and the large number of participants (n = 4139) gave the power to adjust for a range of covariates in the analyses. Fourth, both objective and perceived measures were used to assess the neighbourhood physical environment. The objective neighbourhood environment was assessed with individualized network buffers, giving a better representation of the actual environment than buffers measured through the car network. Finally, environmental perceptions were assessed using a validated questionnaire and two specific physical activity domains were assessed using a validated questionnaire and examined independently.

Besides these strengths, some limitations need to be acknowledged. The most important study limitation was the cross-sectional design, which ruled out the possibility of determining causality. Second, because the response rate was lower than 50% a selection bias towards more motivated women may have been present. Third, women who had moved from the suburbs included in the study were excluded from the analyses because no GIS data could be linked to their addresses. As these women have a less stable location, it might be that mainly the most socio-economically disadvantaged women were over-represented in this group and potentially excluded from the analyses. Fourth, only women between 18 and 46 years were included, so the present results cannot be generalized to a broader population of adult women living in socio-economically disadvantaged neighbourhoods. Finally, because the IPAQ was used to assess physical activity, it remains unclear if the transport-related and leisure-time walking actually took place in the neighbourhood in which the women lived. To clarify this, future studies should include objective measurement methods like Global Positioning Systems (GPS) [24].

## Conclusion

The present study showed that objective physical environmental attributes related to street connectivity and proximity of destinations are positively associated with walking for transportation. This positive association is mainly a direct association, independent on how women perceive the environment. Nonetheless, objectively-assessed street connectivity and proximity of destinations are negatively related to leisure-time walking. This negative association can be explained by the mediating effects of perceived personal safety, aesthetics and neighbourhood social cohesion. Based on these findings, an important message can be formulated for intervention developers and urban planners. It seems that for women living in socio-economically disadvantaged neighbourhoods, a focus on objective environmental characteristics (street connectivity, proximity of destinations) should be accompanied by a focus on improving safety, aesthetics and social cohesion of the neighbourhood, in order to affect both transport-related and leisure-time walking and to achieve larger health effects in the long-term.

## Competing interests

The authors declare that they have no competing interests.

## Authors’ contributions

DVD conducted the statistical analyses and drafted the manuscript. KB coordinated the data collection. JV, LT, IDB and KB participated in the interpretation of the data, helped to draft the manuscript and revised the manuscript for important intellectual content. All authors read and approved the final manuscript.

## References

[B1] Garber CE Blissmer B Deschenes MR Franklin BA Lamonte MJ Lee IM for the American College of Sports Medicine Quantity and quality of exercise for developing and maintaining cardiorespiratory, musculoskeletal, and neuromotor fitness in apparently healthy adults: guidance for prescribing exercise Med Sci Sports Exerc 2011 43 1334 1359 10.1249/MSS.0b013e318213fefb 21694556

[B2] Sallis JF Cervero R Ascher WW Henderson K Kraft MK Kerr J An ecological approach to creating active living communities Ann Rev Publ Health 2006 27 14.1 14.26 10.1146/annurev.publhealth.27.021405.102100 16533119

[B3] Sallis JF Owen N Fisher EB Glanz K, Rimer BK, Viswanath K Ecological models of health behavior Health behavior and health education: theory, research and practice 2008 San Francisco: Jossey-Bass 465 486

[B4] Saelens BE Handy SL Built environment correlates of walking: a review Med Sci Sports Exerc 2008 40 S550 S566 10.1249/MSS.0b013e31817c67a4 18562973 PMC2921187

[B5] Van Holle V Deforche B Van Cauwenberg J Goubert L Maes L Van de Weghe N De Bourdeaudhuij I Relationship between the physical environment and different domains of physical activity in European adults: a systematic review BMC Public Health 2012 12 807 10.1186/1471-2458-12-807 22992438 PMC3507898

[B6] Owen N Humpel N Leslie E Bauman A Sallis JF Understanding environmental influences on walking: review and research agenda Am J Prev Med 2004 27 47 76 10.1016/j.amepre.2004.03.006 15212778

[B7] Bauman AE Reis RS Sallis JF Wells JC Loos RJF Martin BW for the Lancet Physical Activity Series Working Group Correlates of physical activity: why are some people physically active and others not? Lancet 2012 380 258 271 10.1016/S0140-6736(12)60735-1 22818938

[B8] Van Dyck D Cerin E Conway T De Bourdeaudhuij I Owen N Kerr J Cardon G Frank LD Saelens BE Sallis JF Perceived neighborhood environmental attributes associated with adults’ recreational physical activity: findings from the USA, Australia and Belgium Health Place 2013 19 59 68 23178650 10.1016/j.healthplace.2012.09.017

[B9] Gebel K Bauman AE Petticrew M The physical environment and physical activity: a critical appraisal of review articles Am J Prev Med 2007 32 361 369 10.1016/j.amepre.2007.01.020 17478260

[B10] Ball K Brown W Crawford D Who does not gain weight? Prevalence and predictors of weight maintenance in young women Int J Obes 2002 26 1570 1578 10.1038/sj.ijo.0802150 12461673

[B11] King T Kavanagh AM Jolley D Turrell G Crawford D Weight and place: a multilevel cross-sectional survey of area-level social disadvantage and overweight/obesity in Australia Int J Obes 2006 30 281 287 10.1038/sj.ijo.0803176 16331302

[B12] Cleland V Granados A Crawford D Winzenberg T Ball K Effectiveness of interventions to promote physical activity among socio-economically disadvantaged women: a systematic review and meta-analysis Obes Revin press doi:10.1111/j.1467-789X.2012.01058 10.1111/j.1467-789X.2012.01058.x 23107292

[B13] Evenson KR Aytur SA Borodulin K Physical activity beliefs, barriers, and enablers among postpartum women J Womens Health (Larchmt) 2009 12 1925 1934 20044854 10.1089/jwh.2008.1309 PMC2828187

[B14] Ball K Jeffery RW Crawford DA Roberts RJ Salmon J Timperio AF Mismatch between perceived and objective measures of physical activity environments Prev Med 2008 47 294 298 10.1016/j.ypmed.2008.05.001 18544463

[B15] McGinn AP Evenson KR Herrin AH Huston SL Rodriguez DA Exploring associations between physical activity and perceived and objective measures of the built environment J Urb Health 2007 84 162 184 10.1007/s11524-006-9136-4 PMC2231636 17273926

[B16] Dewulf B Neutens T Van Dyck D De Bourdeaudhuij I Van de Weghe N Correspondence between objective and perceived walking times to urban destinations: influence of physical activity, neighbourhood walkability, and socio-demographics Int J Health Geogr 2012 11 43 10.1186/1476-072X-11-43 23046604 PMC3517393

[B17] Van Dyck D Cardon G Deforche B De Bourdeaudhuij I Do adults like living in high-walkable neighborhoods? Associations of walkability parameters with neighbourhood satisfaction and possible mediators Health Place 2011 17 971 977 10.1016/j.healthplace.2011.04.001 21570333

[B18] Kremers SPJ de Bruijn G Visscher TLS van Mechelen W de Vries NK Brug J Environmental influences on energy balance-related behaviors: a dual-process view Int J Behav Nutr Phys Act 2006 3 9 10.1186/1479-5868-3-9 16700907 PMC1481572

[B19] McCormack GR Friedenreich C Sandalack BA Giles-Corti B Doyle-Baker PK Shiell A The relationship between cluster-analysis derived walkability and local recreational and transportation walking among Canadian adults Health Place 2012 18 1079 1087 10.1016/j.healthplace.2012.04.014 22652511

[B20] Manson JE Hu FB Rich-Edwards JW Colditz GA Stampfer MJ Wilet WC Speizer FE Hennekens CH A prospective study of walking as compared with vigorous exercise in the prevention of coronary heart disease in women N Engl J Med 1999 341 650 658 10.1056/NEJM199908263410904 10460816

[B21] Ball K Cleland V Salmon J Timperio AF McNaughton S Thornton L Campbell K Jackson M Baur LA Mishra G Brug J Jeffery RW King A Kawachi I Crawford DA Cohort profile: the Resilience for Eating and Activity Despite Inequality (READI) study Int J Epidemiolin press 10.1093/ije/dys165 PMC4432460 23255533

[B22] Australian Bureau of Statistics Census of Population and Housing: Socio-Economic Indexes for Areas (SEIFA) 2003 Canberra (AUST): ABS

[B23] Dillman DA Mail and telephone surveys: the total design method 1978 New York: Wiley

[B24] Thornton LE Pearce JR Kavanagh AM Using Geographic Information Systems (GIS) to assess the role of the built environment in influencing obesity: a glossary Int J Behav Nutr Phys Act 2011 8 71 10.1186/1479-5868-8-71 21722367 PMC3141619

[B25] Grasser G Van Dyck D Titze S Stronegger W Objectively measured walkability and active transport and weight-related outcomes in adults: a systematic review Int J Public Healthin press doi:10.1007/s00038-012-0435-0 10.1007/s00038-012-0435-0 23224518

[B26] Mujahid MS Diez Roux AV Morenoff JD Raghunathan T Assessing the measurement properties of neighborhood scales: from psychometrics to ecometrics Am J Epidemiol 2007 165 858 867 10.1093/aje/kwm040 17329713

[B27] Sampson RJ Raudenbush SW Earls F Neighborhoods and violent crime: a multilevel study of collective efficacy Sci 1997 277 918 924 10.1126/science.277.5328.918 9252316

[B28] Craig CL Marshall AL Sjöström M Bauman AE Booth ML Ainsworth BE International physical activity questionnaire: 12-country reliability and validity Med Sci Sports Exerc 2003 35 1381 1395 10.1249/01.MSS.0000078924.61453.FB 12900694

[B29] Trost SG Owen N Bauman AE Sallis JF Brown W Correlates of adults’ participation in physical activity: review and update Med Sci Sports Exerc 2002 34 1996 2001 10.1097/00005768-200212000-00020 12471307

[B30] MacKinnon DP Lockwood CM Hoffman JM West SG Sheets V A comparison of methods to test mediation and other intervening variable effects Psychol Methods 2002 7 83 104 11928892 10.1037/1082-989x.7.1.83 PMC2819363

[B31] MacKinnon DP Fairchild AJ Fritz MS Mediation analysis Ann Rev Psychol 2007 58 593 614 10.1146/annurev.psych.58.110405.085542 16968208 PMC2819368

[B32] Parra DC Hoehner CM Hallal PC Ribeiro IC Reis R Brownson RC Pratt M Simoes EJ Perceived environmental correlates of physical activity for leisure and transportation in Curitiba, Brazil Prev Med 2011 52 234 238 21195726 10.1016/j.ypmed.2010.12.008

[B33] Van Dyck D Cardon G Deforche B Giles-Corti B Sallis JF Owen N De Bourdeaudhuij I Environmental and psychosocial correlates of accelerometer-assessed and self-reported physical activity in Belgian adults Int J Behav Med 2011 18 235 245 10.1007/s12529-010-9127-4 21038103

[B34] De Bourdeaudhuij I Teixeira PJ Cardon G Deforche B Environmental and psychosocial correlates of physical activity in Portuguese and Belgian adults Publ Health Nutr 2005 8 886 895 10.1079/phn2005735 16277805

[B35] Saelens BE Sallis JF Frank LD Environmental correlates of walking and cycling: findings from transportation, urban design, and planning literatures Ann Behav Med 2003 25 80 91 10.1207/S15324796ABM2502_03 12704009

[B36] Wendel-Vos W Droomers M Kremers S Brug J van Lenthe F Potential environmental determinants of physical activity in adults: a systematic review Obes Rev 2007 8 425 440 10.1111/j.1467-789X.2007.00370.x 17716300

[B37] Modie-Moroka T Does level of social capital predict perceived health in a community? A study of adult residents of low-income areas of Francistown, Botswana J Health Popul Nutr 2009 27 462 476 19761081 10.3329/jhpn.v27i4.3390 PMC2928095

[B38] Wen M Fan J Jin L Wang G Neighborhood effects on health among migrants and natives in Shanghai, China Health Place 2010 16 452 460 10.1016/j.healthplace.2009.12.001 20060767

[B39] Almedom AM Social capital and mental health: an interdisciplinary review of primary evidence Soc Sci Med 2005 61 943 964 10.1016/j.socscimed.2004.12.025 15955397

